# Transthoracic and transoesophageal echocardiography for tricuspid transcatheter edge-to-edge repair: a step-by-step protocol

**DOI:** 10.1093/ehjimp/qyae017

**Published:** 2024-03-21

**Authors:** Matteo Mazzola, Cristina Giannini, Alessandro Sticchi, Paolo Spontoni, Nicola Riccardo Pugliese, Luna Gargani, Marco De Carlo

**Affiliations:** Department of Surgical, Medical and Molecular Pathology and Critical Care Medicine, University of Pisa, via Paradisa, 2, 56124 Pisa, Italy; Cardiothoracic and Vascular Department, Azienda Ospedaliero-Universitaria Pisana, via Paradisa, 2, 56124 Pisa, Italy; Department of Surgical, Medical and Molecular Pathology and Critical Care Medicine, University of Pisa, via Paradisa, 2, 56124 Pisa, Italy; Cardiothoracic and Vascular Department, Azienda Ospedaliero-Universitaria Pisana, via Paradisa, 2, 56124 Pisa, Italy; Department of Clinical and Experimental Medicine, University of Pisa, Pisa, Italy; Department of Surgical, Medical and Molecular Pathology and Critical Care Medicine, University of Pisa, via Paradisa, 2, 56124 Pisa, Italy; Cardiothoracic and Vascular Department, Azienda Ospedaliero-Universitaria Pisana, via Paradisa, 2, 56124 Pisa, Italy; Department of Surgical, Medical and Molecular Pathology and Critical Care Medicine, University of Pisa, via Paradisa, 2, 56124 Pisa, Italy; Cardiothoracic and Vascular Department, Azienda Ospedaliero-Universitaria Pisana, via Paradisa, 2, 56124 Pisa, Italy

**Keywords:** tricuspid valve, tricuspid regurgitation, transcatheter edge-to-edge repair, transthoracic echocardiography, transoesophageal echocardiography, right ventricle

## Abstract

Tricuspid regurgitation (TR) carries an unfavourable prognosis and often leads to progressive right ventricular (RV) failure. Secondary TR accounts for over 90% of cases and is caused by RV and/or tricuspid annulus dilation, in the setting of left heart disease or pulmonary hypertension. Surgical treatment for isolated TR entails a high operative risk and is seldom performed. Recently, transcatheter edge-to-edge repair (TEER) has emerged as a low-risk alternative treatment in selected patients. Although the experience gained from mitral TEER has paved the way for the technique’s adaptation to the tricuspid valve (TV), its anatomical complexity necessitates precise imaging. To this end, a comprehensive protocol integrating 2D and 3D imaging from both transthoracic echocardiography (TTE) and transoesophageal echocardiography (TOE) plays a crucial role. TTE allows for an initial morphological assessment of the TV, quantification of TR severity, evaluation of biventricular function, and non-invasive haemodynamic evaluation of pulmonary circulation. TOE, conversely, provides a detailed evaluation of TV morphology, enabling precise assessment of TR mechanism and severity, and represents the primary method for determining eligibility for TEER. Once a patient is considered eligible for TEER, TOE, alongside fluoroscopy, will guide the procedure in the catheterization lab. High-quality TOE imaging is crucial for patient selection and to achieve procedural success. The present review examines the roles of TTE and TOE in managing patients with severe TR eligible for TEER, proposing the step-by-step protocol successfully adopted in our centre.

## Graphical Abstract

**Graphical Abstract:**
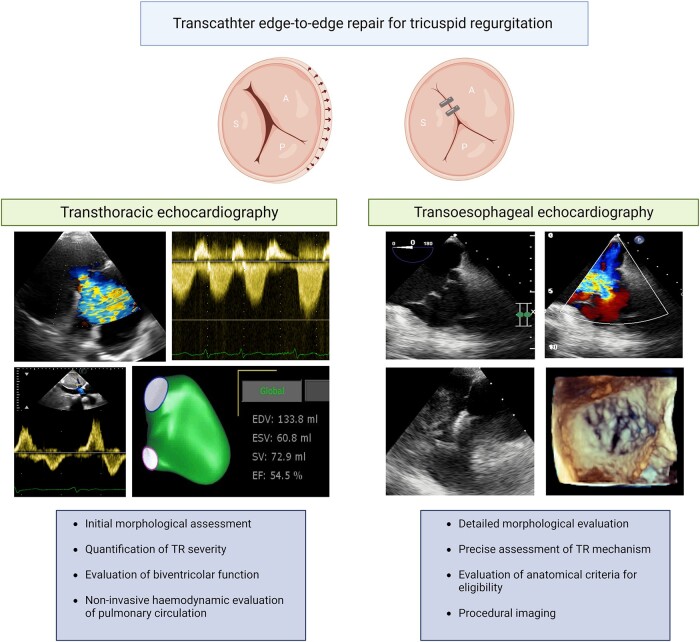
Step-by-step transthoracic and transoesophageal protocol for tricuspid transcatheter edge-to-edge repair.

## Introduction

Tricuspid regurgitation (TR) is the most common disease of the tricuspid valve (TV) and is observed in 0.55% of the general population.^1,2^ Based on the aetiology, TR may be classified as either primary (organic) or secondary (functional).^3^ Primary TR is the least common aetiology of TR and is caused by congenital or acquired intrinsic abnormalities of the leaflet and/or subvalvular apparatus.^3–5^ Secondary TR accounts for more than 90% of cases and results from impaired valve coaptation caused by dilatation of the right ventricle (RV) and/or of the tricuspid annulus (TA).^6^ Given the aging of the population, the rising rates of atrial fibrillation, and the growing trend of intracardiac device implantation, TR is expected to become increasingly prevalent.^7^ Accordingly, the classification of TR based on pathophysiological mechanisms has been recently modified, introducing TR related to Cardiac Implantable Electronic Devices (CIEDs) as a distinct third class.^8^ This presents a significant public health concern due to the independent association of TR with adverse prognosis and the burden of symptoms accompanying progressive right heart failure.^9,10^ The 2022 guidelines from the European Society of Cardiology (ESC) support surgical treatment of TR primarily in combination with left heart surgery or for symptomatic patients with severe TR and RV dilatation, unless severe RV dysfunction is present.^2^ However, surgery for isolated TR remains challenging and is associated with a notable in-hospital mortality rate, primarily because of comorbidities that increase surgical risk.^7^ In this context, a variety of transcatheter treatment strategies have been developed to provide a therapeutic option for high-surgical risk patients with severe TR despite optimal medical therapy. At present, transcatheter edge-to-edge repair (TEER) is the most widely adopted approach, supported by the largest body of evidence.^11^ While experience with TEER of the mitral valve (MV) has facilitated the refinement of this technique for the TV, the anatomical complexity of the TV and the heterogeneity of the disease pose challenges that underscore the critical importance of the correct use of imaging technique.^12^

The aim of this review is to examine the role of transthoracic echocardiography (TTE) and transoesophageal echocardiography (TOE) in the management of patients with severe TR eligible for TEER, proposing the step-by-step protocol that we have successfully implemented at our centre.

### Functional anatomy and pathological changes

TV is the largest of the four cardiac valves with a normal area between 7 and 9 cm^2^ and holds the most anterior and apical position.^12,13^ It is situated between the right atrium (RA) and the RV and serves as one of the two atrioventricular valves, the other being the MV, with which it shares many characteristics.^14^ Despite its complex and variable anatomy, the TV apparatus can be functionally divided into four components, much like the MV: three leaflets (anterior, septal, and posterior), TA, which interacts with the myocardium of the RV and RA, chordae tendineae, and papillary muscles.^12,15,16^

#### The leaflets

**Figure 1:**
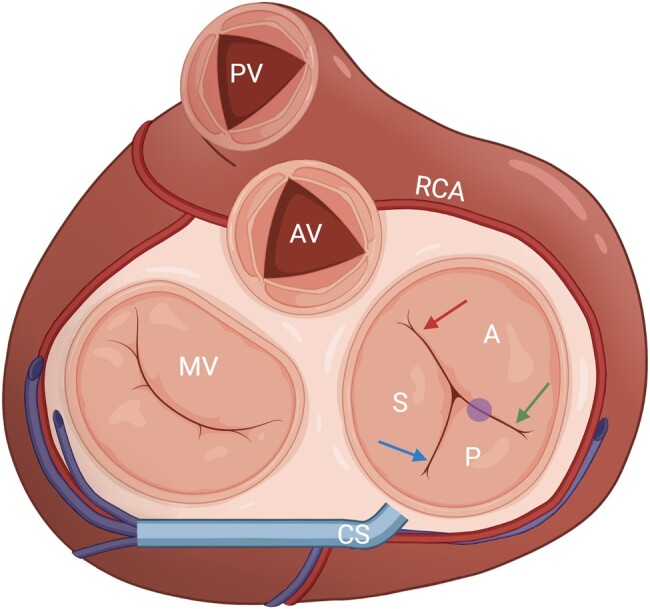
Topographic landmarks of tricuspid valve commissures and leaflets. The anteroseptal commissure (top arrow) is located next to the aortic valve (AV); the posteroseptal commissure (bottom left arrow) is located the level of the coronary sinus (CS) inflow; the anteroposterior commissure (bottom right arrow) is located just above the anterior papillary muscle (circle); the septal leaflet (S) is located between the AV and the CS inflow; the posterior leaflet (P) is located between the CS inflow and the projection of the anterior papillary muscle; the anterior leaflet (A) is located between the projection of the anterior papillary muscle and the anteroseptal commissure. MV, mitral valve; PV, pulmonary valve; RCA, right coronary artery.

**Figure 2:**
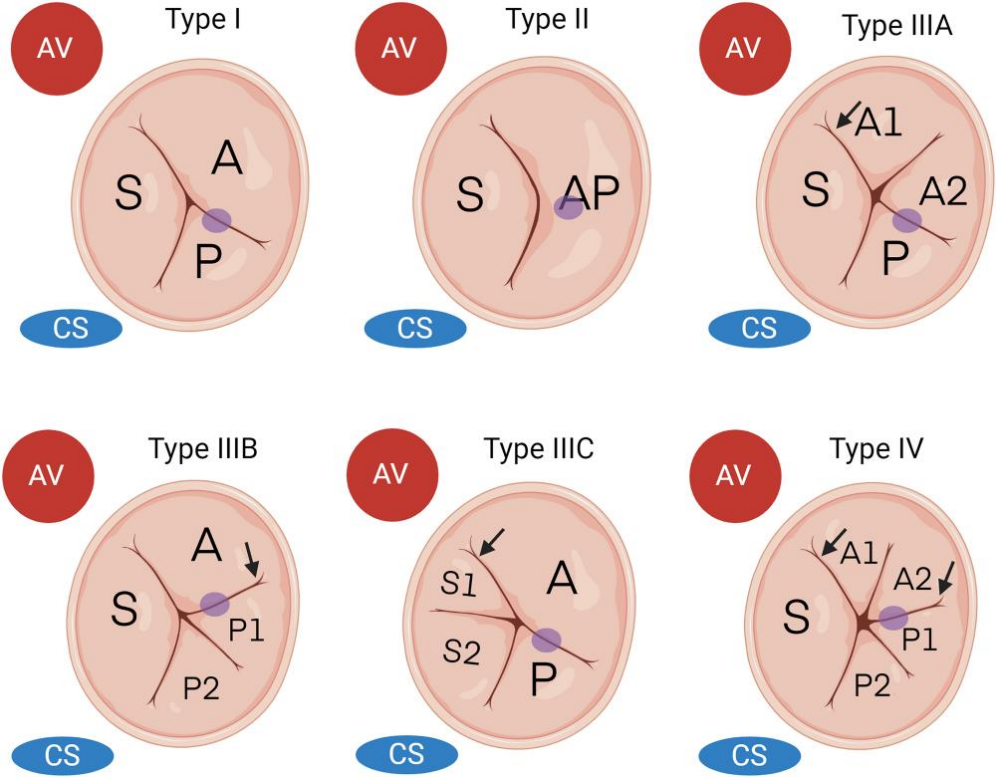
Anatomical variations and nomenclature of the tricuspid valve. The classic tricuspid valve configuration is classified as Type I; the bi-leaflet anatomy is classified as Type II and is characterized by the presence of a distinct septal leaflet (S) along with a large mural leaflet complex (AP); two anterior leaflets (A1, A2) identify type IIIA, with numbering of leaflets starting at the anteroseptal commissure (arrow); two posterior leaflets (P1, P2) identify type III B, with numbering of the leaflets starting at the anteroposterior commissure (arrow); two septal leaflets (S1, S2) identify type IIIC, with numbering of the leaflets starting at the anteroseptal commissure (arrow); the presence of two anterior and two posterior leaflets identifies Type IV, with numbering of the leaflets starting at the anteroseptal and anteroposterior commissures, respectively (arrows). Circle: projection of the anterior papillary muscle. AV, aortic valve; CS, coronary sinus.

TV leaflets display distinctive mechanical properties compared with the MV and can be more challenging to visualize with imaging techniques. Indeed, unlike the MV, the TV leaflets are notably thin, relatively delicate, and less prone to calcification.^4,12,14,15^ The most common configuration of TV consists of three leaflets, which, using anatomical nomenclature based on body position, would be termed antero-superior, inferior, and septal. However, for imaging purposes, they are commonly referred to as anterior, posterior, and septal.^12,15,16^ Within the annulus, the three leaflets are distributed with a circumferential ratio of 1:1:0.75 for the anterior, septal, and posterior leaflet, respectively.^17,18^ The anterior leaflet is the most mobile of the three and displays the greatest radial extension and the largest area.^12,15^ The posterior leaflet, despite having the least circumferential extension, exhibits greater radial length and mobility compared with the septal leaflet.^12,15^ The latter is the least mobile, has the smallest radial extension, and attaches to the septum in a more apical position than the anterior leaflet of the MV, typically not exceeding a distance of 10 mm under physiological conditions.^12,15^ The area where two of these leaflets adhere during RV systole is known as coaptation zone. Normal coaptation takes place at the level of the annulus or just below it, with a coaptation length > 5 mm.^4,10^ The points where two of these leaflets meet and attach to the annulus are called commissures.^15,19^ Due to the considerable anatomical variability, it is not uncommon to encounter variations in the number of leaflets or scallops, and in some cases, the complete absence of a leaflet structure, particularly at the level of anterior and posterior leaflets. Indeed, these two leaflets should be considered as part of a complex mural system encompassing the entire base of the free wall of the RV.^15,16^ However, in the most typical configuration, three distinct commissures can be identified: the anteroseptal, posteroseptal, and anteroposterior.^5,12,15,16^ The use of topographic landmarks to identify these areas is of utmost importance to recognize normal structures as well as anatomic variations and to properly guide transcatheter procedures (*Figure 1*).^5,20,21^ Based on these anatomical landmarks, Hahn *et al*.^20^ have recently introduced a simplified nomenclature for the echocardiographic assessment of TV anatomy, that takes into account the high anatomical variability of the valve (*Figure 2*). In this novel system, derived from the analysis of 579 patients, the anatomical variations of TV are classified according to the number of ‘leaflets’ without making a distinction between true supernumerary leaflets and scallops. This terminology is adopted due to the limitations of echocardiography in making an accurate distinction between these structures and allows for a more comprehensive and practical understanding of TV anatomy. A four-leaflet configuration with an additional posterior leaflet (Type IIIB) was the most common anatomical variation (32.1%) in the population enrolled in the original study.^20^

#### The annulus

**Figure 3:**
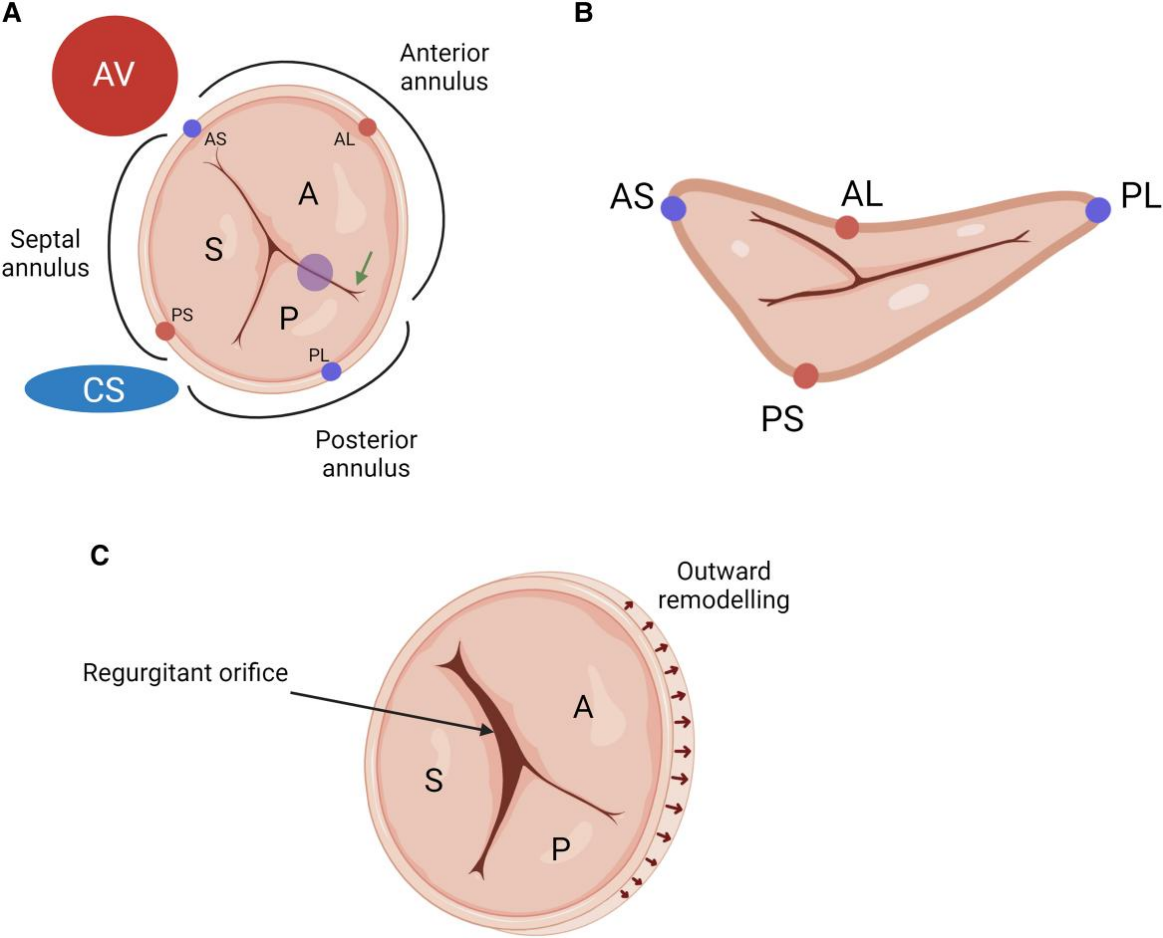
Tricuspid annulus anatomy. (*A*) The anterior annulus is located between the aortic valve (AV) and anteroposterior commissure (arrow); the posterior annulus is located between the anteroposterior commissure and the coronary sinus inflow (CS); the septal annulus is located between the AV and the CS inflow. (*B*) The tricuspid annulus (TA) most superior points (atrially displaced) are the anteroseptal (AS) and the posterolateral (PL) portions (left and right circles); the most inferior points (apically displaced) are the posteroseptal (PS) and the anterolateral (AL) portions (top and bottom circles). (*C*) TA dilation primarily occurs along the anterior and posterior leaflet attachments, causing the annulus to become more circular and planar. As impaired coaptation of the leaflets occurs along the septal coaptation line, the regurgitant orifice is typically crescent-shaped or elliptical.

In contrast to the MV, there is no fibrous continuity between the corresponding semilunar valve and TA, which is not a well-defined anatomical structure but rather represents a functional unit defined anatomically by the atrioventricular (AV) junction and the basal attachment of the TV leaflets to the RV.^15^ The AV junction consists of the juxtaposition of four components: the RA myocardium, the RV myocardium, adipose tissue, and the hinge line of the tricuspid leaflets.^16^ TA is oriented nearly vertically with an ∼45° rotation from the sagittal plane and takes on an asymmetrical, saddle-like ellipsoidal shape^12,15,19^ (*Figure 3*). In healthy individuals, the typical TA area measures 11 ± 2 cm². However, there is a noteworthy increase, reaching 29.6 ± 5.5%, during atrial systole and in the late systole to early diastole phases.^22,23^ The TA can be divided into three areas following the nomenclature of the leaflets and commissures (*Figure 3*).^15^ Ensuring the accurate identification of these areas is critically important for surgical and transcatheter procedural planning, given the close proximity of TA to vital vascular structures. Indeed, the hinge line of the anteroseptal commissure, which is also called aortic segment of TA, is only few millimetres distant form the aortic root. Moreover, the right coronary artery can be as close as 1 mm from the hinge point of the leaflets, particularly in the lower half of the anterior annulus and along the entire posterior annulus.^15,16^

#### Subvalvular apparatus

Similar to the MV, the subvalvular apparatus of the tricuspid valve consists of papillary muscles (PMs) and chordae tendineae.^5,12,15,19,24–26^ However, chordal structures are more fragile compared with the MV, and there are numerous anatomical variations, making the interior of the RV as unique as one’s fingerprint.^5,24^ In the most common configuration, the RV accommodates three types of PMs: anterior, posterior, and septal.^5,24,26^ The largest and most consistently located PM is the anterior, which arises from the anterolateral portion of the RV free wall near the apex and continues with the moderator band. Due to its distinct morphology and consistent presence, the anterior PM serves as a crucial topographic landmark for the anteroposterior commissure.^5,20,26^ The septal PM, also known as the muscle of Lancisi, is the smallest of the PMs and may be absent or negligible in up to 20% of normal patients.^5,24^ For this reason, in contrast to the MV subvalvular apparatus, it is common to find multiple direct chordal attachments from the septum to the septal and anterior tricuspid leaflets.^5,14,24,25^

#### Pathological changes

RV alterations associated with the development of secondary TR can be observed in case of left-sided heart disease (i.e. valve disease or left ventricular dysfunction), pulmonary hypertension (either pre-capillary or post-capillary) and primary RV dilatation or dysfunction (i.e. myocardial disease or RV ischaemia or infarction).^3–5^ Recently, it has been demonstrated that diseases associated with isolated atrial dilatation, such as atrial fibrillation and heart failure with preserved ejection fraction, can also cause TR due to remodelling of the RA and TA, even without RV dilation or dysfunction.^27^ Accordingly, bench-top modelling has suggested that even a modest 40% change in annular dimensions can lead to significant TR in cases of isolated TA dilation, compared with the 75% dilation required for the MV.^28^ Considering these pathophysiological differences, the most recent morphological classification of secondary TR identifies two distinct phenotypes: ‘ventricular functional TR’, associated with RV dilatation or dysfunction, and ‘atrial functional TR’ (formerly known as idiopathic or isolated functional TR), linked to RA and TA dilatation.^3,5^ Morphological abnormalities associated with ventricular functional TR and atrial functional TR are listed in *Table 1*. Regardless of the phenotype, alterations of the TA represent a pivotal mechanism in the development of secondary TR.^28^ Similarly to the intertrigonal portion of the MV annulus, the small septal leaflet is fairly fixed and relatively spared by dilation.^19^ Thus, TA dilation primarily occurs along the anterior and posterior leaflet attachments, causing the annulus to become more circular and planar. As impaired coaptation frequently occurs along the septal coaptation line, regurgitant orifice of secondary TR is typically crescent-shaped or elliptical (*Figure 3*).^3,5^ The classification of TR based on its main pathophysiological mechanisms has recently undergone a modification, introducing TR related to CIEDs as a distinct third class.^29^ The mechanisms leading to CIEDs-related TR have been recently categorized into three main groups: implantation-related, lead-mediated, and pacing-related.^30^ Although evidence is not robust, the technique an operator uses to implant a lead may affect the risk of TV damage. Specifically, employing a ‘prolapsing technique’ appears to increase the likelihood of TR compared with ‘direct crossing’.^30^ Lead-mediated TR (LTR) include the cases where the pacemaker leads interfere with the TV apparatus. This interference can take various forms, such as impinging upon a leaflet, adhering to a leaflet, entangling in the subvalvular apparatus, perforating or lacerating a leaflet, or even avulsion of a leaflet (for instance, during lead extraction procedures).^4,29,30^ In contrast, pacing-related TR related occurs when the RV undergoes enlargement and/or experiences dyssynchrony or dysfunction due to pacemaker stimulation, leading to significant leaflet tethering and/or TA dilation.^4^ Regardless of the mechanism, LTR can be subclassified by echocardiography into Type A and Type B. In Type A, the CIED lead directly causes TR, while in Type B, although a CIED lead is present, it doesn’t directly cause TR but may affect device selection for repair.^8^

**Table 1:** Clinical, morphological, and echocardiographic characteristics of ventricular and atrial functional tricuspid regurgitation

	Ventricular functional TR	Atrial functional TR
Gender	More often male	More often female
Left ventricular ejection fraction	Often reduced	Usually preserved
Left atrium	Mild to moderate dilation	Often severe dilation
Tethering	Often severe	Absent or mild
Tenting height^a^	>9 mm	≤9 mm
Tenting area^a^	≥2.1 cm^2^	<2.1 cm^2^
Tenting volume^a^	≥2.5 mL	<2.5 mL
RA	Mild to moderate dilation	Often severe dilation
End-systolic RA to RV area ratio^a^	<1.5	≥1.5
RV diameter	Severely increased	Normal to mildly increased
RV mid-ventricular diameter^a^	>38 mm	≤38 mm
RV remodelling	Spherical	Preserved conical shape
2D sphericity index^a^	≥55	<55
RV function	Often severely reduced	Normal or mildly reduced
sPAP	Often severely increased (≥50 mmHg)	Normal to mildly increased
Carpentier classification	IIIb	Ib

sPAP, pulmonary arterial systolic pressure; RA, right atrium; RV right ventricle.

^a^See Reference 8 (Hahn *et al. J Am Coll Cardiol 2023*).

### Transthoracic echocardiography

TTE represents the first line imaging in the evaluation of patients with TR who may be candidates for interventional procedures. TTE allows for an initial morphological evaluation of TV. However, given the complexity of its anatomy, it is necessary to complement this information with data obtained from various views.^4,12^ Furthermore, TTE plays a crucial role in quantifying the degree of regurgitation, evaluating the mechanism of TR, assessing RV function, and conducting a non-invasive haemodynamic evaluation of the pulmonary circulation.^13,15^ These aspects are of paramount importance when identifying suitable candidates for TEER. Moreover, TTE serves as the standard method for evaluating systolic and diastolic function of the LV, and disease affecting the MV and AV.^4,31^ This information plays a crucial role, considering that, in the majority of patients with functional TR, the disorder is a consequence of left heart disease. Additionally, it is important to note that in recent clinical trials, a severe reduction in LV systolic function and indications for left-sided valve procedures (e.g. severe aortic stenosis and severe mitral regurgitation) or pulmonary valve correction within the last 60 days represented an exclusion criterion.^11^ Thus, in the absence of any evidence demonstrating the benefits of TEER in these patient cohorts, such conditions need to be thoroughly investigated and therapeutic options individually tailored.

Tips, tricks and avoiding pitfalls for TTE are detailed in the Supplementary data online, *Materials*.

#### Step 1: initial assessment of TV morphology

According to the recommendations of the European Association of Cardiovascular Imaging (EACVI) and the American Society of Echocardiography (ASE), assessing the TV and the RV requires an approach that relies on multiple specific views.^4,31^ The main TTE views allowing the TV visualization are the parasternal long-axis (PLAX) and short-axis views (PSAX), the apical four-chamber (A4C) view and subcostal views (*Table 2*).^4,12,31,32^ However, identifying the leaflets from standard transthoracic views remains a contentious issue, partly because of the variability in imaging planes resulting from varying degrees of transducer angulation, as well as the anatomical variations of the TV.^32^ When possible, the accurate identification of the leaflets enables the localization of the TR jet, the detection of gross primary leaflet abnormalities such as prolapse, vegetations, and perforations and, in some cases, the identification of the position of the lead of CIEDs. TTE 3D imaging allows for a more accurate assessment of TV morphology and is often used in experienced centres to provide a comprehensive evaluation of the TV leaflets, annulus, and subvalvular apparatus.^33^ Although acquisitions of 3D datasets including the TV can be performed from any of the conventional acoustic windows, the apical view represents the most suitable imaging plane to visualize TV leaflets during systole, while for diastolic configuration the parasternal views are preferred.^12,33^ For these reasons, achieving optimal 3D imaging of TV may require positioning the imaging plane between these two standard views. However, capturing multiple volumes from different planes usually provides a more comprehensive assessment of the valve and annulus. Given the complexity of the valve, 3D volume should include adjacent structures to aid in identifying leaflet anatomy.^33^ Using transthoracic TTE, the 3D volume should be manipulated to visualize the TV leaflets from the RV perspective, with the aorta and the anterior leaflet positioned to the right of the screen, and the interatrial septum with the septal leaflet in the far-field at 6 o’clock.^12^

**Table 2:** Transthoracic echocardiography protocol for the tricuspid valve

Anatomy	Echocardiographic view	Description
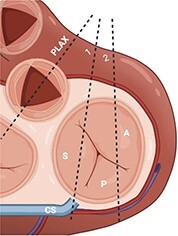	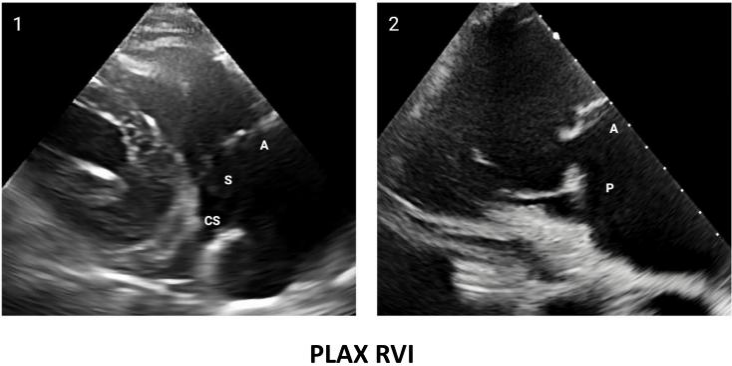	From PLAX, RVI view is obtained by tilting the probe 30° to the right and downward. In this view, A with opposing S or P is visible. When the CS and interventricular septum are in view, A and S are imaged (Box 1). Further rightward and downward tilting removes these structures from the view, enabling visualization of A and P (Box 2) in most cases (77%).
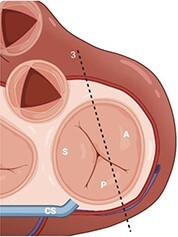	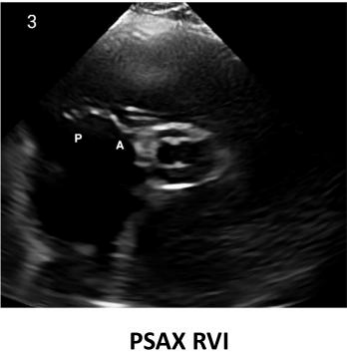	In the PSAX AV view, typically, only the A is visible due to the apical position of the S. When the TA loses its saddle shape due to dilation, both the A (adjacent to the AV) and P (adjacent to the RV free wall) can be imaged (Box 3).
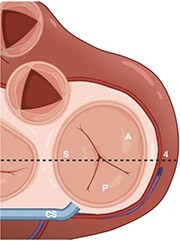	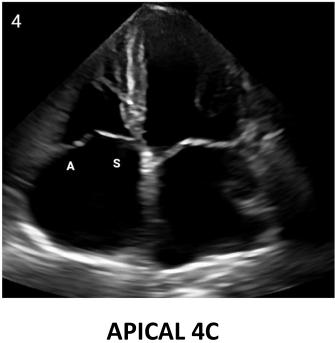	In the apical 4C view (Box 4) of the RV, S is clearly identifiable by using the interventricular septum as landmark. The opposing leaflet is A in 81% of cases. Tilting the probe anteriorly to image AV allows for the clear visualization of A and S. Conversely, P and S become visible with posterior tilting (along with the CS).
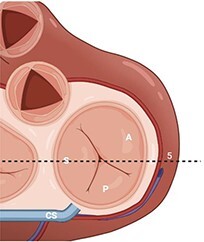	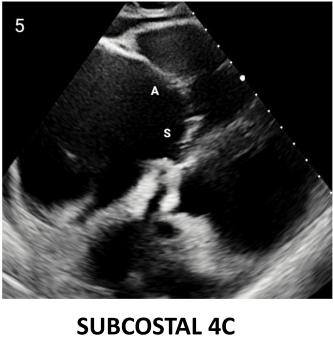	In the subcostal 4C view (Box 5), the leaflets typically observed are the same as in the A4C view (A and S), with variations depending on whether the transducer is tilted anteriorly or posteriorly.
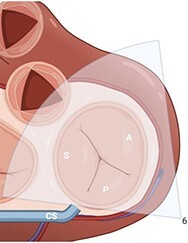	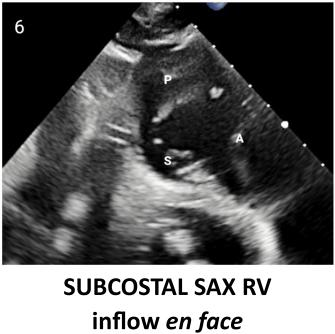	A subcostal SAX RV inflow *en face* view (Box 6) with simultaneous visualization of the three leaflets may be obtained by orienting the transducer along the valve plane at ∼45° angle from the sagittal plane.

A, anterior leaflet; A4C, apical four-chamber; AV, aortic valve; CS, coronary sinus; P, posterior leaflet; PLAX, parasternal long-axis; PSAX, parasternal short-axis; RV, right ventricle; RVI, right ventricle inflow; S, septal leaflet; TA, tricuspid annulus; TV, tricuspid valve.

#### Step 2: grading TR and evaluating the mechanism

TTE is the most employed imaging technique for grading TR. Recommendations from the EACVI and ASE categorize TR as mild, moderate, or severe using qualitative, semi-quantitative, and quantitative measures.^4,34,35^ While this approach shares many principles with the quantification of MR, it is important to note that many of the studies validating the use of these parameters have notable limitations, including a lack of a ‘gold standard’ and insufficient correlation with clinical outcomes.^12^ Qualitative evaluation starts from the morphological assessment of TV, investigating the presence of severe valve lesions such as flail, large coaptation defect and/or severe tenting associated with an elevated likelihood of severe TR.^4,12,36^ The analysis of TR jet with colour Doppler at a Nyquist limit of 50–60 cm/s is mandatory for the detection of TR, but should not be used alone to grade TR.^4,12,13,36^ Colour TR jet is an expression of jet moment rather than a measure of regurgitant volume. Thus, even if a large central jet or an eccentric wall-impinging jet is usually specific for severe TR, patients with elevated pulmonary pressure may demonstrate larger jets leading to an overestimation of TR orifice area. On the other hand, rapid equalization of RA and RV pressures in severe TR may result in a non-aliasing jet, which could mislead the operator.^13^ Similar to the assessment MR, the continuous wave (CW) envelope of the TR jet can serve as a valuable qualitative indicator of TR severity.^34,35^ A dense TR signal with a complete envelope suggests more severe TR, while a faint signal may indicate a milder condition.^4^ In case of severe TR, the CW Doppler envelope may exhibit characteristics such as truncation (notching) with a triangular shape and an early peak velocity (blunting), resulting from elevated RA pressure and/or a prominent regurgitant pressure wave in the RA.^34,35^ Moreover, as the TR orifice area increases, the duration for pressure equalization between the RA and RV shortens. Consequently, the peak velocity of the TR jet on CW tends to decrease with increasing TR severity, frequently falling below 2 m/s in extreme cases.^37^ Qualitative assessment also relies on the analysis of the indirect effects of TR on adjacent structures. The likelihood of severe TR is elevated in presence of a dilated RV with D-shape and/or end-systolic RV eccentricity index > 2, as well as in case of RA area > 18 cm^2^ and inferior vena cava diameter > 21 mm with alteration in respiratory-related collapse,^4,12,31,34–36^ ‘Vena contracta’ (VC) width represents one of the most employed semi-quantitative parameters. VC measurement is usually performed in the A4C view to capture the septo-lateral dimension, considering a minimum of two to three beats (at least 10 in patients with atrial fibrillation). The obtained value in the A4C view should be averaged with the measurement in the RVI view to account the anteroposterior extension of the VC.^38^ A VC value exceeding 7 mm indicates severe TR. It is worth noticing that a VC width < 6 mm is not able to discriminate between mild or moderate TR and, in case of multiple jets, the respective values are not additive.^4^ Moreover, regurgitant orifice of secondary TR are rarely circular, thus 2D VC width may not accurately reflect the severity of TR in case of complex crescent-shape or stellate regurgitant orifice.^4,12^ Since the increased RA pressure due to severe TR affects RV early diastolic filling by increasing RA-RV proto-diastolic gradient, the evaluation of TV E-wave velocity represents a valid semi-quantitative measure with a peak E velocity > 1 m/s suggesting severe TR.^4,34,35^ The transmission of increased RA pressure to the venous system can be assessed by pulsed wave Doppler analysis of hepatic venous flow, which usually demonstrates a progressive reduction of the systolic anterograde flow and provides a semi-quantitative parameter for grading TR. In case of severe TR, an inversion of the hepatic vein systolic flow may be observed with a sensitivity of 80%.^39^ Nevertheless, hepatic vein reversal may be affected not only by TR but also by RA compliance and peak TR pressure gradients, affecting the specificity of this parameter.^8^ Quantitative assessment of TR is mainly based on the analysis of flow convergence area and the application of the proximal isovelocity surface area (PISA) method with a PISA radius > 0.9 cm at a Nyquist limit of 28 cm/s, an effective regurgitant orifice area (EROA) ≥ 40 cm^2^, and regurgitant volume ≥ 45 mL generally denoting severe TR.^4,12,35,36^ However, this method assumes the circularity of the regurgitant orifice and may lead to underestimation of TR by up to 30%, especially for the crescent-shaped and stellate orifices characterizing secondary TR.^40^ The most recent guidelines have expanded the classification to encompass ‘massive’ and ‘torrential’ TR (both of which are associated with unfavourable outcomes) in a new five-grade scheme (*Table 3*).^4^ This expansion in the classification is prompted by the fact that patients undergoing transcatheter tricuspid valve interventions often come for treatment at a late stage in the natural progression of the disease, typically exhibiting an anatomical regurgitant area that is several times larger than 0.40 cm².^41^ Findings from early trials of transcatheter treatment for TR using this grading system indicated that even a slight improvement, such as a one-grade reduction, led to short-term enhancements in patient functional capacity and quality of life.^42,43^ TTE may also provide information to distinguish between the two main morphological forms of TR by assessing TA annulus dimensions, tethering height, tenting area and volume, as well as RA and RV dimensions and function. The presence of severe RA and TA dilation in the absence of RV dilation (RV mid-ventricular diameter ≤ 38 mm and end-systolic RA to RV area ratio ≥ 1.5) and significant tethering of the leaflets is associated with atrial phenotype of functional TR (*Table 1*).^8^ Differentiating between atrial and ventricular phenotypes in functional TR is crucial. Patients with ventricular functional TR, particularly when coupled with pulmonary hypertension and/or left heart disease, have a higher mortality rate than those with atrial functional TR or primary TR.^44,45^

**Table 3:** Grading of severe tricuspid regurgitation (according to the EACVI and ESC council of valvular heart disease position paper^4^)

	Severe	Massive	Torrential
Semi-quantitative parameters VC width (mm)	7–13	14–20	≥21
3D VC area or quantitative Doppler EROA (mm^2^)	75–94	95–114	≥115
Quantitative parameters EROA by PISA (mm^2^)	40–59	60–79	≥80
R Vol (mL)	40–59	60–74	≥75

EROA, effective regurgitant orifice area; PISA, proximal isovelocity surface area; R Vol, regurgitant volume.

Significant annular dilatation is defined by a septo-lateral end-diastolic diameter of ≥40 mm or >21 mm/m^2^ imaged in the A4C of TTE.^4,12^ In the same view, tenting area and tethering height can be measured using an end-systolic frame to grade the tethering of TV leaflets.^8,46,47^

#### Step 3: assessing RV and pulmonary circulation

According to current ESC guidelines on valvular heart disease, severe RV dysfunction and the presence of severe pulmonary hypertension are considered contraindications for interventional treatment of TR, even though clear intervention thresholds are lacking.^2^ To properly assess candidates for TEER, a comprehensive evaluation of RV dimensions and function, as well as an analysis of pulmonary circulation, should be performed in all severe TR patients.^2,13^ While a multimodality imaging protocol, including cardiac magnetic resonance (CMR) and/or cardiac computed tomography, along with right heart catheterization (RHC), is regarded as the gold standard for evaluating the RV-pulmonary circulation unit, TTE serves as the primary and easily accessible imaging tool.^2,13,16,48^ For the assessment of RV dysfunction, the commonly used M-mode and 2D TTE functional parameters include: RV fractional area change < 35%, tricuspid annular plane systolic excursion (TAPSE) < 17 mm, systolic myocardial velocity < 9.5 cm/s, and free-wall longitudinal strain < 20%.^31^ 2D TTE methods, less influenced by load, are more accurate in predicting events and identifying RV dysfunction than TAPSE alone, which should not be used in isolation for RV functional assessment.^13^ Moreover, recent use of 3D TTE has enabled global RV systolic function assessment by calculating the RV ejection fraction (RVEF), showing a good correlation with cardiac CMR values.^31^ In functional TR patients undergoing TEER, RVEF < 44.6% has been identified as a robust predictor of post-intervention survival.^49^ Systolic function parameters, while sophisticated, do not account for the relationship between RV contractility and the afterload of the pulmonary circulation, which is estimated in terms of right ventricular–arterial coupling (RVPAc). Although this parameter is typically measured through RHC, the ratio between TAPSE and systolic pulmonary artery pressure (sPAP) derived from TTE has been demonstrated to be correlated with the invasive gold-standard measures.^50^ Moreover, RV-PA uncoupling, defined as a TAPSE/sPAP ratio < 0.406 mm/mmHg, has proved to be an independent significant predictor of 1-year all-cause mortality following transcatheter TEER for severe TR.^51^ However, in patients with severe TR, echocardiographic estimation of sPAP based on the peak TR velocity (TRV) should be interpreted cautiously. In the most severe cases, due to the rapid equalization of atrial and ventricular pressures, TRV tends to decrease, leading to an underestimation of echocardiographic sPAP.^4^ This disparity between echocardiographic sPAP and the invasive gold-standard has recently been examined in a population of patients with severe TR undergoing TEER, revealing a higher incidence of post-procedure events in those with a difference between the two values exceeding 10 mmHg.^52^ This finding underscores the importance of having information about pulmonary haemodynamics before performing TEER. From the analysis of RHCs before TEER, a haemodynamic profile characterized by pulmonary hypertension (mean PAP > 30 mmHg) with features suggestive of pre-capillary involvement (transpulmonary gradient > 17 mmHg) has been associated with higher 1-year mortality.^53^ Moreover, by combining RHC parameters with echocardiographic assessment of RV-PA coupling and analysing the secondary TR phenotype, clinicians can take informed decisions regarding transcatheter treatment of concurrent severe mitral and tricuspid regurgitation. In these cases, patients with severe or greater TR and suitable anatomy for TV TEER, especially those exhibiting an atrial-predominant phenotype and preserved RV-PA coupling, without pulmonary hypertension or with isolated post-capillary pulmonary hypertension, may significantly benefit from a combined transcatheter approach addressing both TR and mitral regurgitation.^54^

### Transoesophageal echocardiography

TOE is an invaluable imaging tool for the assessment of patients with severe TR.^5^ In patients with good acoustic windows, TOE provides a comprehensive evaluation of TV morphology, enabling a precise assessment of the degree and mechanism of TR.^4,5,13,36^ Furthermore, TOE is the most used method for determining TV repair feasibility and selecting among interventions like TEER, annuloplasty, TV replacement, or lead extraction. Effective decision-making regarding the best approach relies on a thorough understanding of valve anatomy and TR mechanism and requires collaboration within the heart valve team.^5,8,13,36,55^ Moreover, TOE, along with fluoroscopy, is utilized to guide the procedure in the catheterization lab.^21^ Consequently, the ability to provide high-quality TOE images has become a crucial criterion in patient selection for TEER.^13,56^

Tips, tricks and avoiding pitfalls for TOE are detailed in the Supplementary data online, *Materials*.

#### Step 1: comprehensive morphological assessment

**Figure 4:**
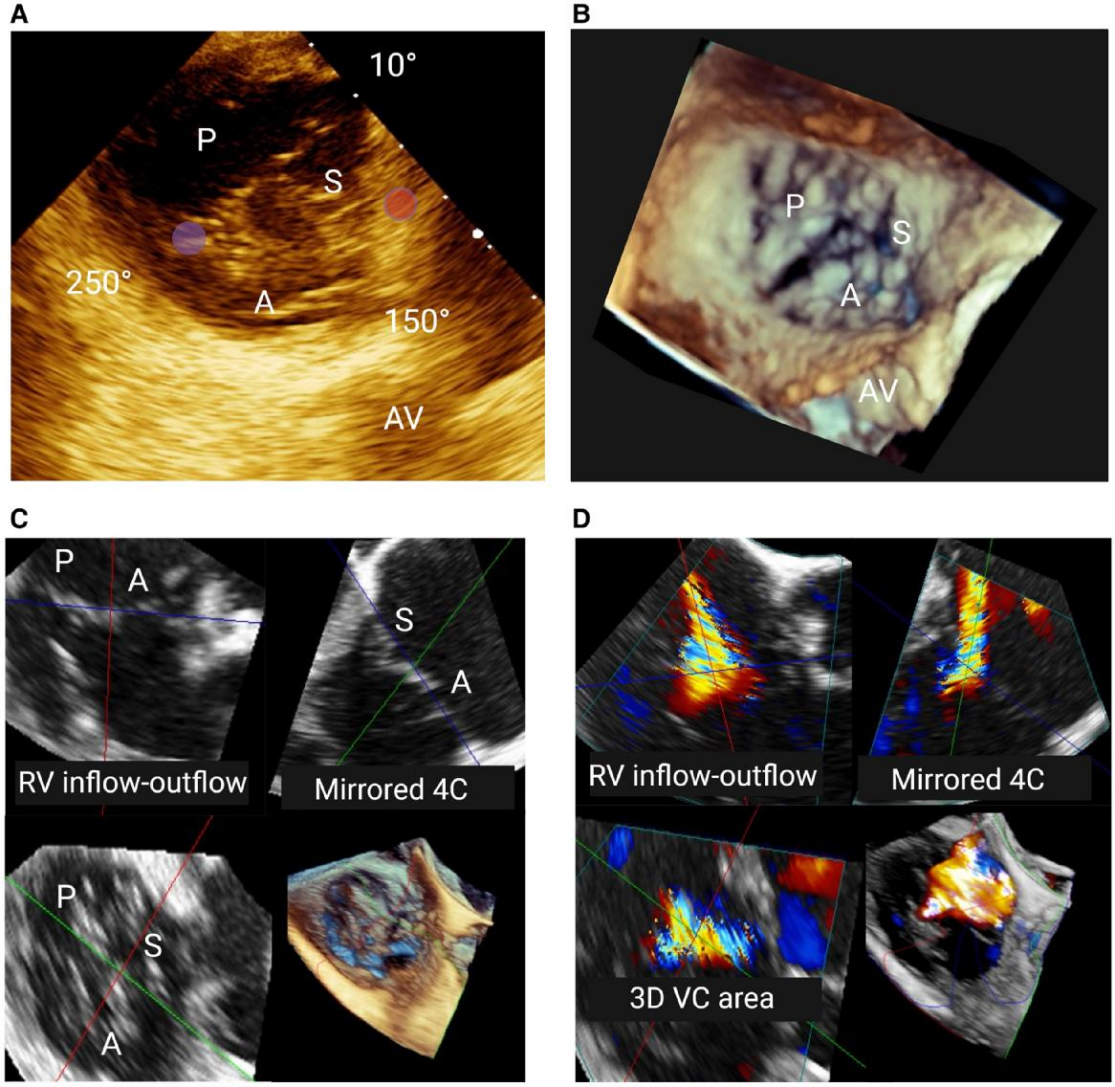
Transgastric short-axis view of tricuspid valve (TV) and 3D volume. (*A*) The anteroseptal commissure is adjacent to the aortic valve (AV) at the 150° position; a slight advancement of the scanning level usually reveals the anterior papillary muscle (left circle), which identifies the anteroposterior commissure, usually at the 250° position; the interventricular septum (right circle) is displayed on the right side of the screen and identifies the septal leaflet, while the posteroseptal commissure is at the junction between the septum and the posterior wall, generally corresponding to the 10° position. (*B*) To ease interpretation, the 3D volume of the TV should be rotated so that the septum appears on the right side of the screen, with the AV at 5 o’clock, replicating the orientation of the TV in the transgastric short-axis view. (*C* and *D*) Multiplanar reconstruction of a 3D dataset without (*C*) and with colour (*D*). AV, aortic valve; 4C, four-chamber view; RV, right ventricle; VC, vena contracta.

The apical and anterior position of TV, the complex annulus morphology, and large valve area pose a challenge for imaging with TOE requiring a multi-level scanning approach to thoroughly assess its structure. Main imaging levels include: mid-oesophageal (ME) views, deep-oesophageal (DE) views and transgastric (TG) view (*Table 4*). DE is a new imaging level introduced in the most recent ASE recommendations for TOE in screening for structural heart interventions and is particularly relevant due to the close proximity of the TV plane to the gastroesophageal junction.^5^ From DE plane, advancing the probe into the stomach and applying a gentle anteflexion of the probe results in the visualization of the TG scanning plane. Adjusting the mechanical rotation to a range of 25–60° enables the visualization of the short-axis (SAX) TG view of the TV, which simultaneously displays all three leaflets.^5,13,55,56^ This view is pivotal for TEER as it enables the precise localization of leaflets and commissures, assessment of the regurgitant jets position, and examination of any potential coaptation gap.^5,13,55,56^ Moreover, in patients with CIEDs, TG view allows for a clear visualization of the lead crossing point and the interaction between the lead and the leaflets.^57^ In this view, the TV is observed *en face*, and the localization of leaflets and commissures relies on previously mentioned anatomical landmarks (*Figure 4*).^20,57,58^ From the TG view, Advancing the TOE probe further into the stomach with rightward anterior flexion results in a deep-transgastric view of the TV, which allows for optimal colour Doppler evaluation of TR jets.^5,12^ The high complexity of the valvular and subvalvular apparatus frequently requires the use of 3D imaging to ensure a precise morphological assessment. Similar to TTE, data acquisition for 3D imaging can be performed in any scanning plane. However, the 0° DE projection is particularly useful for 3D reconstruction, as the DE view at 0° avoids interference from structures of the left heart.^5,12,55^ Using 3D TOE, the 3D volume is rotated to visualize the structure from the atrial side. Specific orientation of 3D volume is displayed in *Figure 4*. In cases of CIEDs-related TR, the use of 3D TOE enables characterization of the lead position and its relationship with the leaflets, especially when TG view is suboptimal. In this case, the application of multiplanar reconstruction (MPR) to 3D TOE allows for the reconstruction of this standard 2D TOE views from a single 3D dataset (*Figure 4*).^57^

**Table 4:** Transoesophageal echocardiography protocol for the tricuspid valve

Anatomy	Echocardiographic view	Description
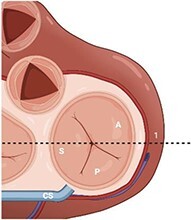	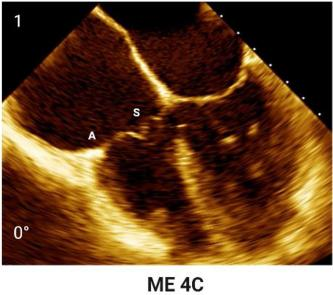	Starting from ME level, ME4C view is imaged at ∼0° of rotation. Typically, the S and A are visible (Box 1).
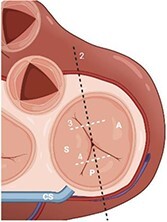	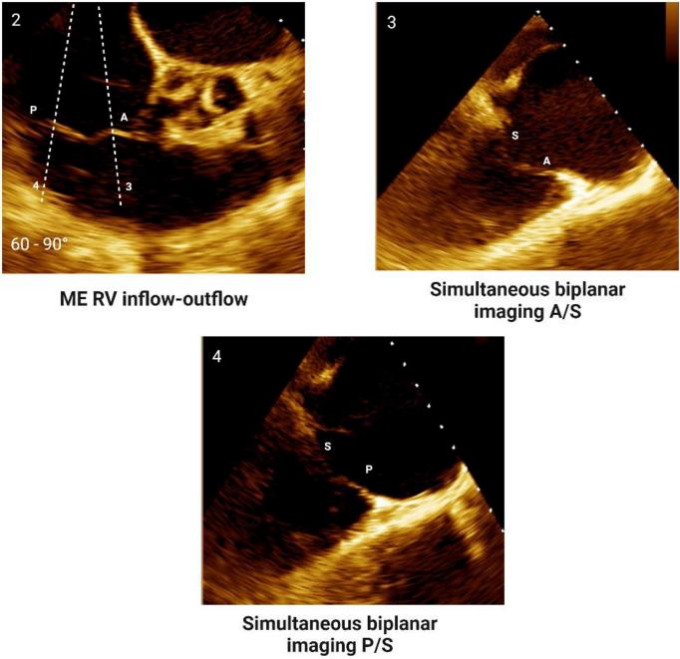	Changing the degree of rotation to 60° shows the ME RV inflow–outflow view, where A is displayed near the AV and P is in the opposite position (Box 2). Simultaneous biplanar imaging should be acquired scanning from the aortic side to the posterior annulus in order to visualize the anteroseptal and posteroseptal commissure lines (Box 3–4). ‘Tips and Tricks Box’. When the imaging plane is correct, activating biplane imaging generates a mirrored ME 4C. A quality marker for imaging is full visualization of S without shadowing artefacts, particularly at the A/S commissure (Box 3).
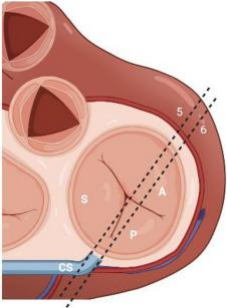	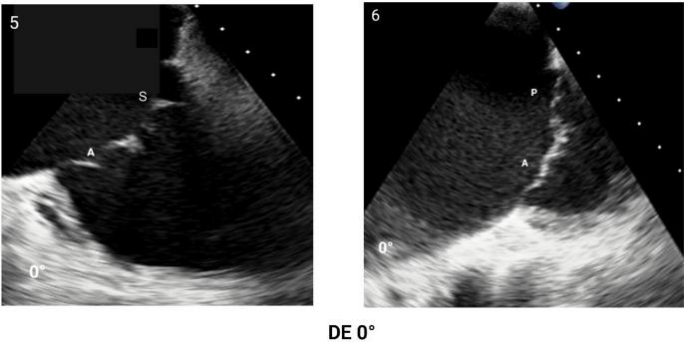	From the ME plane, advancing the probe into the distal oesophagus allows for the visualizing of the DE scanning plane. At 0° of mechanical rotation, this scanning plane excludes the left atrium and instead offers a clear view of RA, TV, CS inflow and RV. In this view, A is typically visible with opposing the S (Box 5) or P, if a gentle retroflexion is applied (Box 6).
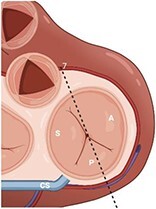	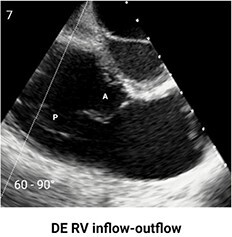	Adjusting the degree of mechanical rotation to 60–90° provides a view of the DE RV inflow–outflow (Box 7). In this view, simultaneous biplanar imaging can be employed, as previously described for the ME plane. ‘Tips and Tricks Box’. The correct search angle of TOE transducer might be higher than the angle for the ME RV inflow–outflow view (up to 80–100°).
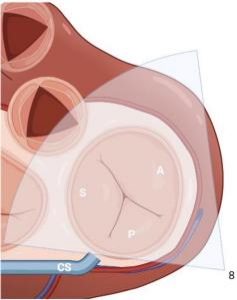	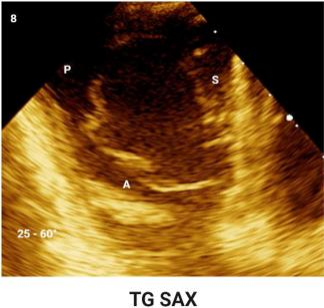	From DE plane, advancing the probe into the stomach and turning to the right allows for the visualization of TG imaging planes. Adjusting the mechanical rotation to a range of 25–60° enables the visualization of the SAX TG view (Box 8) of the TV, which simultaneously displays all three leaflets. ‘Tips and Tricks Box’. Higher angles might be necessary for cases of significant RV enlargement and cardiac axis deviation. The primary orientation of TG imaging can be adjusted using simultaneous biplane imaging, ensuring a vertical alignment of the TA on the secondary imaging plane (see Supplementary data online, *Video S3*).

A, anterior leaflet; CS, coronary sinus; DE, deep-oesophageal; ME, mid-oesophageal; ME4C, mid-oesophageal four-chamber; RA, right atrium; RV, right ventricle; S, septal leaflet; SAX, short-axis; TG, transgastric; TV, tricuspid valve.

#### Step 2: grading TR and assessing the mechanism

The grading of TR as well as the identification of the main mechanism with TOE follows the same principles as TTE, with the advantage of obtaining higher definition 2D and 3D images. The application of MPR to 3D images obtained through TOE mitigates the limitations of bidimensional assessments in estimating the severity of TR.^4,12^ MPR of colour Doppler 3D enables the identification of the regurgitant jet in various scanning planes within the 3D volume. By aligning the axes at the point corresponding to the VC, 3D VC area can be measured by manual planimetry (*Figure 4*). A value > 40 mm² denotes a severe regurgitant jet, but data correlating this parameter with prognosis are still lacking.^4^ This technique proves especially valuable in cases of multiple jets and eccentric regurgitant orifices, overcoming the limitations of 2D VC.^4,12^ Moreover, MPR reconstruction of TA allows for the calculation of 3D planimetry annular area, which can be used along with a pulsed sample volume at the annulus to calculate diastolic stroke volume. Subtracting the forward stroke volume, obtainable from either the left or right ventricular outflow tract, provides the regurgitant volume of TR.^12^ However, these methods necessitate validation to ensure their accuracy and reliability.

#### Step 3: assessing the eligibility for TEER

Morpho-functional analysis performed with TOE plays a pivotal role in identifying patients whose anatomy is favourable for TEER (*Table 5*). The best procedural outcomes are achieved for tri-leaflet TV configuration with regurgitant jets located at the anteroseptal commissure and small-sized septo-lateral coaptation gap (≤7 mm).^13^ However, newer-generation devices with different implant sizes have been demonstrated to provide favourable procedural outcomes even for septo-lateral coaptation gaps of ≤8.4 mm.^59^ The location of the coaptation gap has also been demonstrated to impact procedural success. Non-central or non-anteroseptal jets are associated with poorer procedural outcomes and an increased risk of residual TR post-repair. Conversely, treating isolated septoanterior coaptation defects is associated with much more favourable results.^60,61^ Distinguishing between the gap size at the target zone for device implantation and the maximum gap size at the centre of the valve is crucial. The former directly impacts the likelihood of successful device implantation, whereas the latter influences the overall success probability of the device.^57^

**Table 5:** Assessment of eligibility for tricuspid transcatheter edge-to-edge repair

	Favourable	Feasible	Unfavourable
Anatomical criteria	Coaptation gap ≤ 7 mm	Coaptation gap between 7 and 8.5 mm	Coaptation gap ≥ 8.5 mm
	Anteroseptal jet	Posteroseptal jet	Anteroposterior jet
	Confined prolapse or flail	Wide prolapse or flail	Multiple prolapses or flails
	Tri-leaflet morphology	Anatomic variations	Severe alterations of the leaflets
	No CIED lead	CIED lead without impingement	CIED lead with impingement
	Favourable angle of approach	Feasible angle of approach	Unfavourable angle of approach
Right ventricular function and pulmonary pressure	Normal to moderately reduced RV function, normal to moderate RV remodelling	Moderately reduced RV function, moderate RV remodelling	Severely reduced RV function and/or severe RV remodelling
	No PH or mildly increased pulmonary pressures	Mild to moderate isolated post-capillary PH	Severe pulmonary hypertension and/or pre-capillary PH
Quality of imaging	Good TOE windows	Sufficient TOE windows	Insufficient TOE windows

CIED, cardiac implantable electronic devices; PH, pulmonary hypertension; RV, right ventricle; TOE, transoesophageal echocardiography.

Leads from CIEDs can complicate the ability to perform TEER because of the interference with delivery system trajectory and the impingement of the leaflets.^13^ TOE enables the visualization of lead position and the assessment of its relationship with the leaflets. The positioning of the lead is crucial for patients with CIEDs-related TR undergoing TEER of the TV. Improved procedural outcomes (TR grade reduction ≤ 1) are observed when the leads cross the TV plane centrally or commissurally, as opposed to crossing at the level of the leaflet body.^62^ Moreover, if there is interference between the lead and the trajectory of delivery system, and after ensuring there is no leaflet impingement, an evaluation of lead mobility can be conducted using steerable catheters under fluoroscopic and TOE guidance.^63^ These manoeuvres are performed to assess the feasibility of positioning the lead away from the delivery system’s trajectory, thus allowing for a TEER procedure free from interference. This approach has been recently suggested for patients with CIEDs and leads in place for at least three months.^63^ However, the evidence supporting this technique is solely based on individual clinical cases, and further studies are required to assess effectiveness and safety.

#### Step 4: guiding the procedure

TV TEER aims to restore proper coaptation between valve leaflets and reduce the regurgitant orifice. This procedure targets the commissures, typically favouring an anteroseptal clipping approach,^15,61^ which has been associated to better post-procedural haemodynamics in *ex vivo* model.^61^ However, the choice of the target zone and the number of devices depend closely on valve anatomy and on jet location (*Table 6*).^64^ Guidance for TEER involves effective communication between the interventional cardiologist and echocardiographer.^21^ The initial placement of the guidewire and navigation of the clip delivery system into the RA are optimally visualized through ME bi-caval view, ME modified bi-caval view (100–110°), or ME four-chamber view with use of simultaneous biplane imaging (see Supplementary data online, *Video S1*).^21,56^ Upon reaching the TA, when the clip is still in the RA, comprehensive visualization of the septal leaflet, clip device trajectory adjustment, target zone localization, and preliminary clip rotation are best achieved using either the ME or DE RV inflow–outflow views, complemented by simultaneous biplane imaging (see Supplementary data online, *Video S2*).^55^ For the final clip rotation, the TG SAX view offers an all-encompassing perspective, revealing all three leaflets during coaptation.^13,21,55,56^ Advancement of the clip into the RV should be performed in this view with simultaneous biplanar imaging for continuous monitoring of clip rotation (see Supplementary data online, *Video S3*). After ensuring proper positioning, the clip arms are oriented perpendicular to the coaptation line using ME or DE RV inflow–outflow view. In this projection, when the clip is correctly aligned, the scanning plane is positioned perpendicular to the clip’s arms, so they do not appear in the image. Simultaneous biplanar imaging provides a scanning plane parallel to the clip’s arms, allowing them to be seen in the image (see Supplementary data online, *Video S4*).^36,55^ A gradual retraction of the clip towards the atrium allows the leaflets to delicately settle on the clip arms. To ascertain satisfactory leaflet grasping, the primary view is the ME or DE RV inflow–outflow, with supplementary assistance from the multiplane view to monitor both clip arms and leaflets (see Supplementary data online, *Video S5*).^13,21,55,56^ Before releasing the clip, it is crucial to confirm adequate leaflet grasping by means of multiple views to ensure restricted leaflet motion on 2D imaging and the presence of an adequate tissue bridge on 3D imaging.^21,55^ After clip deployment, the final evaluation should include assessing TV inflow gradient (TV mean pressure gradient < 3 mmHg) and residual TR severity (reduction ≥ 1 grade) (see Supplementary data online, *Videos S6* and *S7*).^55^

**Table 6:** Multiclip strategies for tricuspid transcatheter edge-to-edge repair

Strategy^a^	Technique	Advantages
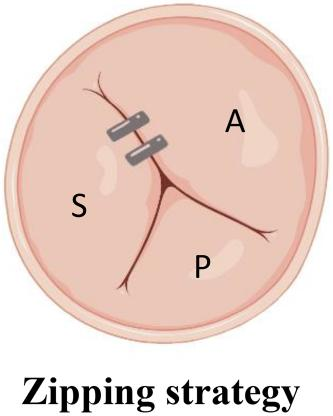	Anteroseptal approach Devices are strategically positioned moving from the commissure towards the centre of the valve.	Superior post-procedural haemodynamics according to *ex vivo* model^54^
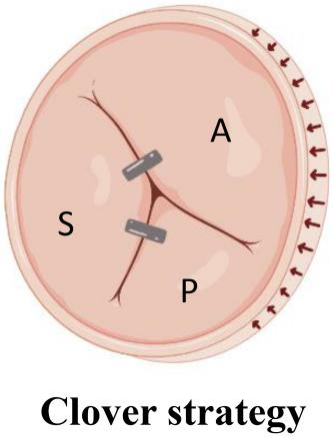	A triple orifice TV is created by approaching both the anteroseptal and posteroseptal commissures	Inward pull force on the TA and RV free wall counteracting the typical septo-mural dilation of the TA

A, anterior leaflet; P, posterior leaflet; RV, right ventricle; S, septal leaflet; TA, tricuspid annulus; TV, tricuspid valve.

^a^The images are displayed from atrial perspective, with the aortic valve positioned at 11 o’clock.

## Conclusions

Given the complex anatomy of the TV, a comprehensive protocol integrating 2D and 3D imaging from both TTE and TOE assumes a paramount role for TEER. These imaging approaches serve as indispensable tools from screening to intraprocedural guidance, ensuring informed decision-making, and enhancing patient outcomes. Ongoing research efforts are essential to continually improve these techniques and integrate them with other advanced imaging methods.

## Supplementary Material

qyae017_Supplementary_Data

## Data Availability

No new data were generated or analysed in support of this research.

## Supplementary data

Supplementary data are available at *European Heart Journal – Imaging Methods and Practice* online. **Consent:** Written informed consent was obtained from the patients whose echocardiographic images and videos are included in the manuscript.

## Lead author biography

Marco De Carlo, MD, PhD, FESC I obtained the degree in Cardiology and the PhD summa cum laude at Pisa University. I am the director of Interventional Cardiology at Pisa University Hospital and Associate Professor of Cardiology at Pisa University. I authored over 190 scientific papers, including 3 European Guidelines, with an h-index of 44. My areas of interest include coronary, structural, and peripheral interventions. I served as Chairperson of the Working Group on Aorta and Peripheral Vascular Diseases of the European Society of Cardiology, and as member of the Congress Programme Committee.
