# Correction to: Transthoracic and transoesophageal echocardiography for tricuspid transcatheter edge-to-edge repair: a step-by-step protocol

**DOI:** 10.1093/ehjimp/qyae068

**Published:** 2024-07-16

This is a correction to: Matteo Mazzola, Cristina Giannini, Alessandro Sticchi, Paolo Spontoni, Nicola Riccardo Pugliese, Luna Gargani, Marco De Carlo, Transthoracic and transoesophageal echocardiography for tricuspid transcatheter edge-to-edge repair: a step-by-step protocol, *European Heart Journal - Imaging Methods and Practice*, Volume 2, Issue 2, January 2024, qyae017, https://doi.org/10.1093/ehjimp/qyae017

Due to an error, this paper was incorrectly published in Volume 2 Issue 1 of the journal. It has now been republished in Volume 2 Issue 2 as part of the special issue on Valvular Heart Disease.

