# Supplementary material for: Transthoracic and transoesophageal echocardiography for tricuspid transcatheter edge-to-edge repair: a step-by-step protocol
Source: Eur Heart J Imaging Methods Pract. 2024 Mar 21;2(2):qyae017. doi: 10.1093/ehjimp/qyae017 (PMC11195804; doi:10.1093/ehjimp/qyae017)
Supplement: qyae017_Supplementary_Data [file qyae017_supplementary_data.zip › TV.TEER.SupplementalMaterials.CLEAN.docx]

**Supplemental materials**

**Tips, tricks and avoiding pitfalls for transthoracic echocardiography** ………………………... 2

**Tips, tricks and avoiding pitfalls for transoesophageal echocardiography** …………………… 5

**Supplemental Videos** ……………………………………………………………………………… 7

**References** …………………………………………………………………………………………. 8

**Tips, tricks and avoiding pitfalls for transthoracic echocardiography**

Considering the complex and variable anatomy of the TV, a multi-window approach is essential during 2D TTE to ensure a reliable mental 3D reconstruction of TV geometry, particularly when dealing with shadowing artifacts from surrounding (e.g., prosthetic valves) or external (e.g., pericardial calcifications) structures.^1,2^ However, subtle variations in transducer position, angulation, or rotation can significantly alter the appearance of the TV in 2D TTE, rendering this modality sometimes insufficient for accurate leaflet identification and morphological evaluation. Subcostal SAX of the RV at the level of the TV (**Table 2**) is the only 2D TTE view that enables the contemporary visualization of all the leaflets in a single cross-sectional imaging plane, improving the localization of the regurgitant jet and the analysis of the interaction between leaflets and pacing lead in patients with CIEDs.^1,3^ Unfortunately, obtaining this view may be unfeasible in cases with challenging subcostal windows, and leaflet visualization is limited to the ventricular perspective.^3,4^ The integration of 3D imaging in TTE approach demonstrates a feasibility of 90% in patients with adequate 2D images and overcomes many of the limitations associated with the exclusive use of 2D TTE.^5,6^ 3D TTE offers a comprehensive assessment of the TV from both atrial and ventricular perspectives, providing a clear en face view that encompasses all leaflets.^6^ Moreover, although current recommendations only support the use of diastolic diameter in 2D echocardiography for the evaluation of the TA, 3D TTE allows for a more accurate assessment of the shape and dimensions of this complex and dynamic structure.^6^ While enhancing the morphological evaluation of TV, this imaging method presents notable limitations in discerning tissue characteristics, such as the presence of calcifications, fibrosis, or vegetations.^4,6^ Additionally, assessing leaflet thickness poses difficulties, as 3D imaging often exhibits leaflets as thicker than their true dimensions due to blurring and/or amplification artifacts.^4,6^

Since the presence of the lead in patients with CIEDs can induce imaging artifacts and signal attenuation during TTE colour flow mapping, performing a TOE in cases of CIEDs-related TR is always essential to accurately determine severity, thus preventing underestimation of the grading.^2^

According to current guidelines, the recommended parameters for TR grading largely mirror those employed for assessing mitral regurgitation.^2^ This recommendation persists despite accumulating evidence that underscores intrinsic differences specific to TV including leaflet number, orifice size, geometry, and the unique pathophysiology of functional TR. The multiparametric and hierarchical approach endorsed by imaging guidelines, incorporating qualitative, semi-quantitative, and quantitative parameters, partially mitigates the inherent limitations of single echocardiographic measures and has demonstrated good performance compared to CMR.^7,8^ Nevertheless, to ensure a reliable evaluation of TR severity, considerations such as respiratory variability, volume load dependency and responsiveness to diuretic therapy should be taken into account.^7^ Indeed, in cases of severe TR, the worsening of TR during inspiration leads to an increase in RA pressure, closely approximating RV systolic pressure. Notably, a TRV decrease of ≥ 0.6 m/s is often observed during inspiration in severe TR, impacting EROA calculations.^9^ To address these dynamic variations, echocardiographic measurements should be acquired ideally at end-expiration in spontaneously breathing patients, when alveolar pressure is near zero.^7,9^ Furthermore, comprehensive TR assessment is recommended when patients are euvolemic, on stable diuretic therapy, with optimized pulmonary pressures, and maintaining normal systemic blood pressure.^7^

Given the prognostic significance of pulmonary hemodynamic assessment, echocardiographic evaluation should include parameters accurately reflecting invasive measurements. However, as previously mentioned, echocardiographic estimation of sPAP can be significantly affected in patients with severe TR.^1,2^ Utilizing non-invasive hemodynamic estimates independent of TRV can overcome these limitations, providing a comprehensive evaluation of pulmonary pressures and RV-PA coupling. Doppler assessment of pulmonary regurgitation enables measurement of peak regurgitation velocity and end-diastolic regurgitation velocity, providing accurate estimates of mean and diastolic pulmonary pressures, respectively.^10^ Moreover, diastolic dysfunction assessment and detection and quantification of lung ultrasound for B-lines can be used to identify those patients with increased pulmonary arterial wedge pressure and extravascular lung water.^11,12^ Finally, although TAPSE/sPAP is associated with worse outcomes among patients undergoing TV TEER, RV-PA coupling can also be estimated using RV stroke volume indexed to RV end-systolic volume measured by 3D TTE.^7,13^ This approach has recently been associated with significant prognostic implications in patients with moderate and severe TR.^13^

**Tips, tricks and avoiding pitfalls for transoesophageal echocardiography**

The assessment of the coaptation line at the commissural level is a crucial aspect in pre-procedural planning for TV TEER. In this context, RV inflow-outflow views, acquired at the ME or DE levels (**Table 4**), correspond to the "commissural" view of the MV. When employed as primary views, simultaneous multiplane imaging from the posterior TV annulus towards the aorta, commonly known as "sweep imaging", captures the coaptation site along the entire septal leaflet.^14-16^ This technique facilitates the localization of the regurgitant jet. If ME imaging quality is suboptimal, using DE, due to its proximity to the TA, ensures an optimal alignment of the ultrasound beam, enhancing both multiplane imaging for sweeping across the TA plane and CW Doppler evaluation of the TR jet.^14,16^

Intraprocedural guidance remains a significant challenge, restricting access to TV TEER. Therefore, when assessing eligibility, it is crucial to confirm adequate imaging quality of the TV in a supine patient position, before considering TEER.^15^ During the procedure, while the RV inflow-outflow views (ME or DE) are typically considered pivotal for leaflet grasping visualization, the TG SAX view of the TV usually offers an effective alternative. This view not only aids in visualizing device introduction into the RV but can also facilitate leaflet grasping (known as the *Mainz-Approach*). TG SAX provides several morphological information of the TV, making it easy to identify critical features like commissures or indentations between leaflets/scallops that need to be avoided during grasping. Moreover, the TG view enables a precise imaging of the device, leaflet engagement, and insertion into the device, thanks to the numerous and precise information provided by a transversal imaging plane and reduced shadowing artifacts. In cases where shadowing artifacts in ME and DE views hinder ME and DE views, the use of TG views becomes decisive for treatment success.^17^

Throughout the procedure, frequent probe adjustments hinder the operator's ability to maintain proper orientation, elevating the risk of oesophageal damage. Consequently, the utilization of 3D live MPR may represent a significant tool for intraprocedural imaging, enabling the reconstruction of standard 2D TOE views from a single 3D dataset (**Figure 4**). Additionally, in the presence of acoustic shadowing from nearby structures, alternative views can be adopted, and 3D live MPR can be applied to replicate the desired 2D views.^18^ Especially in those cases with suboptimal 2D imaging, live MPR can be also useful for guiding leaflet grasping.^17^ To ensure an accurate 3D MPR evaluation of TV morphology, proper alignment of all three imaging planes is crucial. One plane should align parallel to the TV annulus, creating a transversal view. The orientation of the other two planes should establish a RV inflow-outflow view and a 4C view, respectively (**Figure 4**).^17^

In patients with unsatisfactory TOE image quality, 2D (and four-dimensional) intracardiac echocardiography (ICE) emerges as a valuable imaging modality.^16,19^ It provides high-resolution, real-time visualization of cardiac structures, continuous monitoring of catheter location within the heart, and early detection of procedural complications. ICE has already demonstrated its advantage for operators performing tricuspid TEER in selected cases with unfavourable TOE image quality.^19^

**Supplemental Videos**

**Supplemental Video 1.** Guiding the navigation of the delivery system in the right atrium using mid-oesophageal bi-caval view with simultaneous biplanar imaging.

**Supplemental Video 2.** Optimization of the position of the delivery system with respect to the regurgitant jet before entering the ventricle, using mid-oesophageal right ventricular inflow-outflow view with simultaneous biplanar imaging and colour Doppler.

**Supplemental Video 3.** Monitoring the advancement and “clocking” of the device (i.e. perpendicular orientation with respect to the target commissure) in the right ventricle using the transgastric short-axis view with simultaneous biplanar imaging.

**Supplemental Video 4.** Mid-oesophageal right ventricular inflow-outflow view with simultaneous biplanar imaging confirming the correct “clocking” of the clip before leaflet grasping.

**Supplemental Video 5.** Mid-oesophageal RV inflow-outflow view with simultaneous biplanar imaging showing adequate grasping of anterior and septal leaflets.

**Supplemental Video 6.** After deployment of the first clip, the severity of regurgitation is reassessed, and a second device is deployed in the anteroseptal commissure. Right ventricular inflow-outflow view with simultaneous biplanar imaging is used to control the correct positioning of the second clip next to the first one.

**Supplemental Video 7**. Transgastric short-axis displaying the final result of the procedure with a mild residual tricuspid regurgitation.

**References**

1. Hahn RT. State-of-the-Art Review of Echocardiographic Imaging in the Evaluation and Treatment of Functional Tricuspid Regurgitation. *Circ Cardiovasc Imaging* 2016;9. <https://10.1161/CIRCIMAGING.116.005332>

2. Lancellotti P, Pibarot P, Chambers J, La Canna G, Pepi M, Dulgheru R, et al. Multi-modality imaging assessment of native valvular regurgitation: an EACVI and ESC council of valvular heart disease position paper. *Eur Heart J Cardiovasc Imaging* 2022;23:e171-e232. <https://10.1093/ehjci/jeab253>

3. Stankovic I, Daraban AM, Jasaityte R, Neskovic AN, Claus P, Voigt JU. Incremental value of the en face view of the tricuspid valve by two-dimensional and three-dimensional echocardiography for accurate identification of tricuspid valve leaflets. *J Am Soc Echocardiogr* 2014;27:376-384. <https://10.1016/j.echo.2013.12.017>

4. Faletra FF, Leo LA, Paiocchi VL, Schlossbauer SA, Borruso MG, Pedrazzini G, et al. Imaging-based tricuspid valve anatomy by computed tomography, magnetic resonance imaging, two and three-dimensional echocardiography: correlation with anatomic specimen. *Eur Heart J Cardiovasc Imaging* 2019;20:1-13. <https://10.1093/ehjci/jey136>

5. Mediratta A, Addetia K, Yamat M, Moss JD, Nayak HM, Burke MC, et al. 3D echocardiographic location of implantable device leads and mechanism of associated tricuspid regurgitation. *JACC Cardiovasc Imaging* 2014;7:337-347. <https://10.1016/j.jcmg.2013.11.007>

6. Muraru D, Hahn RT, Soliman OI, Faletra FF, Basso C, Badano LP. 3-Dimensional Echocardiography in Imaging the Tricuspid Valve. *JACC Cardiovasc Imaging* 2019;12:500-515. <https://10.1016/j.jcmg.2018.10.035>

7. Hahn RT, Lawlor MK, Davidson CJ, Badhwar V, Sannino A, Spitzer E, et al. Tricuspid Valve Academic Research Consortium Definitions for Tricuspid Regurgitation and Trial Endpoints. *J Am Coll Cardiol* 2023;82:1711-1735. <https://10.1016/j.jacc.2023.08.008>

8. Zhan Y, Senapati A, Vejpongsa P, Xu J, Shah DJ, Nagueh SF. Comparison of Echocardiographic Assessment of Tricuspid Regurgitation Against Cardiovascular Magnetic Resonance. *JACC Cardiovasc Imaging* 2020;13:1461-1471. <https://10.1016/j.jcmg.2020.01.008>

9. Sade LE, Muraru D, Marsan NA, Agricola E, Stankovic I, Donal E. How to assess severe tricuspid regurgitation by echocardiography? *Eur Heart J Cardiovasc Imaging* 2022;23:1273-1276. <https://10.1093/ehjci/jeac015>

10. Abbas AE, Fortuin FD, Schiller NB, Appleton CP, Moreno CA, Lester SJ. Echocardiographic determination of mean pulmonary artery pressure. *Am J Cardiol* 2003;92:1373-1376. <https://10.1016/j.amjcard.2003.08.037>

11. Nagueh SF, Smiseth OA, Appleton CP, Byrd BF, 3rd, Dokainish H, Edvardsen T, et al. Recommendations for the Evaluation of Left Ventricular Diastolic Function by Echocardiography: An Update from the American Society of Echocardiography and the European Association of Cardiovascular Imaging. *J Am Soc Echocardiogr* 2016;29:277-314. <https://10.1016/j.echo.2016.01.011>

12. Gargani L, Girerd N, Platz E, Pellicori P, Stankovic I, Palazzuoli A, et al. Lung ultrasound in acute and chronic heart failure: a clinical consensus statement of the European Association of Cardiovascular Imaging (EACVI). *Eur Heart J Cardiovasc Imaging* 2023;24:1569-1582. <https://10.1093/ehjci/jead169>

13. Gavazzoni M, Badano LP, Cascella A, Heilbron F, Tomaselli M, Caravita S, et al. Clinical Value of a Novel Three-Dimensional Echocardiography-Derived Index of Right Ventricle-Pulmonary Artery Coupling in Tricuspid Regurgitation. *J Am Soc Echocardiogr* 2023;36:1154-1166.e1153. <https://10.1016/j.echo.2023.06.014>

14. Hahn RT, Saric M, Faletra FF, Garg R, Gillam LD, Horton K, et al. Recommended Standards for the Performance of Transesophageal Echocardiographic Screening for Structural Heart Intervention: From the American Society of Echocardiography. *J Am Soc Echocardiogr* 2022;35:1-76. <https://10.1016/j.echo.2021.07.006>

15. Hahn RT, Nabauer M, Zuber M, Nazif TM, Hausleiter J, Taramasso M, et al. Intraprocedural Imaging of Transcatheter Tricuspid Valve Interventions. *JACC Cardiovasc Imaging* 2019;12:532-553. <https://10.1016/j.jcmg.2018.07.034>

16. Hungerford SL, Rye EE, Hansen PS, Bhindi R, Choong C. Key Echocardiographic Considerations for Tricuspid Valve Transcatheter Edge-to-Edge Repair. *J Am Soc Echocardiogr* 2023;36:366-380 e361. <https://10.1016/j.echo.2023.01.013>

17. da Rocha ESJG, Ruf TF, Hell MM, Tamm A, Geyer M, Munzel T, et al. Transgastric imaging-The key to successful periprocedural TEE guiding for edge-to-edge repair of the tricuspid valve. *Echocardiography* 2021;38:1948-1958. <https://10.1111/echo.15196>

18. Wollborn J, Schuler A, Sheu RD, Shook DC, Nyman CB. Real-Time Multiplanar Reconstruction Imaging Using 3-Dimensional Transesophageal Echocardiography in Structural Heart Interventions. *J Cardiothorac Vasc Anesth* 2023;37:570-581. <https://10.1053/j.jvca.2022.11.011>

19. Wong I, Chui ASF, Wong CY, Chan KT, Lee MK. Complimentary Role of ICE and TEE During Transcatheter Edge-to-Edge Tricuspid Valve Repair With TriClip G4. *JACC Cardiovasc Interv* 2022;15:562-563. <https://10.1016/j.jcin.2021.12.035>
